# New mouse models for metabolic bone diseases generated by genome-wide ENU mutagenesis

**DOI:** 10.1007/s00335-012-9397-z

**Published:** 2012-04-21

**Authors:** Sibylle Sabrautzki, Isabel Rubio-Aliaga, Wolfgang Hans, Helmut Fuchs, Birgit Rathkolb, Julia Calzada-Wack, Christian M. Cohrs, Matthias Klaften, Hartwig Seedorf, Sebastian Eck, Ana Benet-Pagès, Jack Favor, Irene Esposito, Tim M. Strom, Eckhard Wolf, Bettina Lorenz-Depiereux, Martin Hrabě de Angelis

**Affiliations:** 1 Institute of Experimental Genetics, Helmholtz Zentrum München, German Research Center for Environmental Health (GmbH), Ingolstaedter Landstr. 1, 85764 Neuherberg, Germany; 2 Chair for Molecular Animal Breeding and Biotechnology, Gene Center of the Ludwig-Maximilians-Universität München, Ludwig-Maximilians-Universität München, Feodor-Lynen-Str. 25, 81377 Munich, Germany; 3 Institute of Pathology, Helmholtz Zentrum München, German Research Center for Environmental Health (GmbH), Ingolstaedter Landstr. 1, 85764 Neuherberg, Germany; 4 Department of Prosthetic Dentistry, University Medical Center, Hamburg-Eppendorf, Martinistr. 52, 20246 Hamburg, Germany; 5 Institute of Human Genetics, Helmholtz Zentrum München, German Research Center for Environmental Health (GmbH), Ingolstaedter Landstr. 1, 85764 Neuherberg, Germany; 6 Institute of Pathology, Technische Universität München, Ismaningerstr. 22, 81675 Munich, Germany; 7 Lehrstuhl für Experimentelle Genetik, Technische Universität München, 85350 Freising-Weihenstephan, Germany; 8 Present Address: Karlsruher Institut fuer Technologie, DE Innovationsmanagement (IMA), Hermann-von-Helmholtz-Platz 1, 76344 Eggenstein-Leopoldshafen, Germany

## Abstract

Metabolic bone disorders arise as primary diseases or may be secondary due to a multitude of organ malfunctions. Animal models are required to understand the molecular mechanisms responsible for the imbalances of bone metabolism in disturbed bone mineralization diseases. Here we present the isolation of mutant mouse models for metabolic bone diseases by phenotyping blood parameters that target bone turnover within the large-scale genome-wide Munich ENU Mutagenesis Project. A screening panel of three clinical parameters, also commonly used as biochemical markers in patients with metabolic bone diseases, was chosen. Total alkaline phosphatase activity and total calcium and inorganic phosphate levels in plasma samples of F1 offspring produced from ENU-mutagenized C3HeB/FeJ male mice were measured. Screening of 9,540 mice led to the identification of 257 phenodeviants of which 190 were tested by genetic confirmation crosses. Seventy-one new dominant mutant lines showing alterations of at least one of the biochemical parameters of interest were confirmed. Fifteen mutations among three genes (*Phex*, *Casr*, and *Alpl*) have been identified by positional-candidate gene approaches and one mutation of the *Asgr1* gene, which was identified by next-generation sequencing. All new mutant mouse lines are offered as a resource for the scientific community.

## Introduction

Metabolic bone diseases originate from endocrine dysfunctions as well as from heterogeneous determinants, including age, life style, and environmental influences. Bone turnover is physiologically regulated by hormones, cytokines, and growth factors and is under the control of numerous signaling pathways (Chavassieux et al. 2007). Metabolic diseases may have primary or secondary impact on bone mineralization. For investigating disease development and progression and to understand the underlying mechanisms, mice have been shown to serve successfully as model organisms (e.g., Abe et al. 2007; Kurima et al. 2002; Marklund et al. 2010; McGowan et al. 2008). Random *N*-ethyl-*N*-nitrosourea (ENU) mutagenesis is a promising approach to obtain mouse models for inherited human diseases (Hrabě de Angelis and Balling 1998). This has been shown in worldwide ENU mutagenesis programs, including bone metabolism, using dual-energy X-ray absorptiometry (DEXA), X-ray analysis, biochemical markers, or the SHIRPA protocol for the phenotyping of ENU mutagenesis-derived C3H/HeJ, BALB/cCRLAnn, and B57BL/6 J mice (Barbaric et al. 2008; Smits et al. 2010; Srivastava et al. 2003).

Within the large-scale Munich ENU mutagenesis screen more than 850 mutant mouse lines have been isolated, derived from a large-scale genome-wide screen (Hrabě de Angelis et al. 2000) or from an implemented modifier screen on *Dll1* ^*lacZ*^ knockout mice (Rubio-Aliaga et al. 2007). Our Dysmorphology Screen is focusing on the isolation of new mouse models for hereditary metabolic bone diseases (Fuchs et al. 2006; Lisse et al. 2008).

In previous studies in mice the reliability of biochemical markers for skeletal disorders, including alkaline phosphatase (ALP), has been shown (Srivastava et al. 2001). Combined ALP, total calcium (Ca), and inorganic phosphate (P_i_) measurements in serum or plasma are routinely performed in patients with metabolic bone diseases (Table 1).

**Table 1:** Mouse models for metabolic bone diseases with altered ALP activity and Ca and P_i_ values in plasma and corresponding human diseases with equivalent changes

Mouse models	*ALP	Ca	P_i_	Human disease
^A^Fgf23^R176Q^	↑^a^	Ø^a^	↓^a^	Autosomal dominant hypophosphatemic rickets (ADHR)
^B^Dmp1^tm1.1Mis^, ^C^DMP1^tm1Jqf^	↑^b^	Ø^b^	↓^c^	Autosomal recessive hypophosphatemic rickets (ARHR)
^D^Slc34a3^tm1Kimi^	↑^d, e^	Ø^f, g^	↓^e, g^	Hereditary hypophosphatemic rickets with hypercalciuria (HHRH)
^E^Phex^Hyp−2J^, ^E^Phex^Hyp-Duk^, ^F^Phex^Hyp^ Gy, Ska1, Pug, ^L^BAP012, ^L^BAP024	↑↑^h^	Ø	↓^b^	X-linked hypophosphatemic rickets (XLHR)
^G^Alpl^Hpp^, ^H^Alpl^tm1(cre)Nagy^, ^I^Alpl^tm1Jlm^, ^K^Alpl^tm1Sor^, ^L^BAP020, ^L^BAP023, ^L^BAP026, ^L^BAP027, ^L^BAP032	↓^h, i^	↑^k^	Ø-↑^k^	Hypophosphatasia (HPP)
e.g., ^M^Col1a1^Aga2^, ^N^Col1a2^oim^, ^O^Col1a1^Mov13^, ^P^Col1a1^Tm1Jae^	↑^l, m^	Ø	n	Osteogenesis imperfecta
^Q^Vcp^tm1Igl^	↑↑^i^	Ø^n^-↑^o^	Ø^p^	Paget’s disease of bone (PDB)
^R^Casr^tm1Ces^, ^L^BCH002, ^L^BCH003, ^L^BCH004, ^L^BCH007, ^L^BCH011, ^L^BCH013, ^L^BCH014	Ø	↑^q,r^	↓^q,r^	Primary hyperparathyroidism

*Ø* unchanged, *↑* increased, *↓* decreased, *n* no data, *ALP* alkaline phosphatase, *Ca* total calcium, *P*_*i*_ total inorganic phosphate

^a^Econs and McEnery (1997), ^b^Lorenz-Depiereux et al. (2006a), ^c^Lorenz-Depiereux et al. (2010), ^d^Mejia-Gaviria et al. (2010), ^e^Lorenz-Depiereux et al. (2006b), ^f^Delmas (1992), ^g^Tieder et al. (1985), ^h^Mornet et al. (2001), ^i^Whyte (2010), ^k^Chodirker et al. (1990), ^l^Cundy et al. (2007), ^m^Braga et al. (2004), ^n^Singer et al. (1998), ^o^Freeman (1988), ^p^Goseki-Sone et al. (2005), ^q^Bilezikian et al. (2005), ^r^Tiosano and Hochberg (2009)

^A^Farrow et al. (2011), ^B^Feng et al. (2008), ^C^Feng et al. (2003), ^D^Segawa et al. (2009), ^E^Lorenz-Depiereux et al. (2004), ^F^Eicher and Southard (1972), ^G^Hough et al. (2007), ^H^Lomeli et al. (2000), ^I^Narisawa et al. (1997), ^K^MacGregor et al. (1995), ^L^Table 4, ^M^Lisse et al. (2008), ^N^Chipman et al. (1993), ^O^Bonadio et al. (1990), ^P^Liu (1995), ^Q^Badadani et al. (2010), ^R^Ho et al. (1995)

Ca and P_i_ homeostasis is balanced by intestinal absorption, mobilization, or binding in bone and renal excretion. Ca levels directly and indirectly influence intestinal phosphate absorption. Much less is known about the influences on P_i_ homeostasis (Bergwitz and Jüppner 2010). A key role in maintaining phosphate homeostasis is the reabsorption of phosphate from urine into the renal proximal tubules. A previously identified phosphaturic factor, FGF23 (fibroblast growth factor 23), acts as an endocrine hormone on the regulation of P_i_ reabsorption in the kidney and on renal vitamin D metabolism (ADHR Consortium 2000; Strom and Jüppner 2008).

Here we describe the results of the Munich ENU Mutagenesis Project to obtain new mutant mouse models for impaired bone metabolism by phenotyping for alterations of at least one of the described plasma parameters as selection markers. We isolated 71 new murine models that may be of special value for the development of new therapeutic approaches since a high number of metabolic bone diseases in human patients are caused by point mutations (Marini et al. 2010; Simon-Bouy et al. 2008; Wenkert et al. 2011).

## Material and methods

### Mice

For this study we used C3HeB/FeJ (C3H) inbred mice purchased originally from the Jackson Laboratory (Bar Harbor, ME, USA) and bred in our animal facility. The mice were housed and handled according to the federal animal welfare guidelines and the state ethics committee approved all animal studies. The mice were kept in a 12/12-h dark–light cycle and provided standard chow ad libitum (TPT total pathogen-free chow #1314: calcium content 0.9 %, phosphate 0.7 %, vitamin D3 600 IE; Altromin, Lage, Germany) and water. Hygienic monitoring was performed following FELASA recommendations (Nicklas et al. 2002). Mutant mouse lines derived from our screen were given internal lab codes and were assigned with official gene symbols and names after the mutation was identified.

### ENU mutagenesis

ENU mutagenesis treatment of inbred strain C3H males was as described previously (Aigner et al. 2011). Litters produced from the ENU-treated C3H males (G0) are designated F1 in the following, while offspring produced from confirmed mutant F1 animals are designated G2.

### Generation of F1 mice and confirmation of phenotypes in a dominant breeding strategy

The F1 animals investigated for this study were derived from a total of 893 G0 males from 15 different ENU-treated groups. Blood samples of 9,540 F1 animals (4,606 females and 4,934 males) were screened for alterations of total ALP, Ca, and P_i_ blood plasma levels. F1 mice showing alterations of blood-based parameters were retested after 14 days. Breeding for confirmation of a dominant phenotype was performed as described previously (Aigner et al. 2007).

### Blood measurements

Blood samples (250 μl) were obtained from 12-week-old nonfasted anesthetized mice by puncture of the retro-orbital sinus, as already described (Rathkolb et al. 2000). All samples were collected between 9:00 and 11:00 a.m. Plasma analysis of ALP, Ca, and P_i_ was done using an Olympus AU400 autoanalyzer (Olympus, Hamburg, Germany) and adapted test kits (Klempt et al. 2006). Descriptive data are expressed as mean ± standard deviation. PTH values were analyzed with a Mouse Intact PTH ELISA Kit (TECOmedical, Bünde, Germany).

### DXA and X-ray measurement

DXA (pDEXA Sabre, Norland Medical Systems Inc., Basingstoke, Hampshire UK, distributed by Stratec Medizintechnik GmbH, Pforzheim, Germany) and X-ray (Faxitron, Hewlett Packard, Palo Alto, CA, USA) measurements were performed for in-depth analysis in selected mouse lines as described previously (Abe et al. 2006; Fuchs et al. 2011).

### Genetic mapping

To map the mutations, ENU-derived mutant mice were outcrossed to wild-type C57BL/6 J (B6) mice, as described previously (Aigner et al. 2009). For linkage analysis, SNP (single-nucleotide polymorphism) genotyping by high-throughput MALDI-TOF (matrix-assisted laser desorption/ionization time-of-flight) technology supplied by Sequenom (San Diego, CA, USA) was performed with a panel containing 158 markers evenly distributed over the whole genome (Klaften and Hrabě de Angelis 2005). We developed the internal MyGenotype database for statistical SNP data analysis.

### Mutation analysis

*Casr*, *Phex*, *Alpl*, and *Asgr1* exons were amplified with intronic primers and directly sequenced using BigDye v3.1 cycle sequencing (Applied Biosystems, Life Technologies, Foster City, CA, USA). *Casr* consists of 7 exons (NM013803), *Phex* (NM011077) consists of 22 exons, and *Alpl* (NM007431) consists of 12 exons. All primer sequences are available upon request. The mutation of the BAP005 mutant line was detected by chromosome sorting (CHROMBIOS, Raubling, Germany) and whole-chromosome sequencing on a Genome Analyzer IIx (Illumina, San Diego, CA, USA). DNA extraction from sorted chromosomes 11 was performed overnight at 42 °C with 0.25 M EDTA, 10 % Na lauroyl sarcosine, and 50 μg proteinase K. Extracted DNA was precipitated and resuspended in TE buffer. Paired-end libraries were constructed with the Illumina paired-end DNA sample preparation kit according to the manufacturer’s protocols and as described previously (Eck et al. 2009). Alignment of the reads was performed with the BWA software, and subsequent analysis was performed with the SAMtools package. In total, ~82 million reads and ~157 million reads were generated for the mutant and control strain, respectively, of which 64 % mapped to the target chromosome 11 for the mutant strain, while 26 % of the control strain reads were on target. The identified nonsynonymous sequence variation in *Asgr1* was confirmed in mutant mice by capillary sequencing.

### Statistical analysis

Statistical analysis of parameters of F1 animals and sex- and age-matched wild-type C3H mice were performed using the software package JMP Release 5.1 (SAS Institute, Cary, NC, USA). The reference values were obtained from untreated age-matched C3H wild-type control groups (50 males and 50 females). Single F1 variants for ALP activity and Ca levels were defined by a *Z* score ≥3 or ≤–3 compared to the age-matched control groups. Mice showing hypophosphatemia were tested three times to confirm P_i_ changes. A *Z* score of ≤–2 was taken to select variants for hypophosphatemia. Statistical differences (*P* values) of the means of ALP, Ca, or P_i_ blood values between all tested affected mice and nonaffected littermates of a mutant line were assessed by one-way analysis of variance (ANOVA), *t* test (giving mean ± SD values), and the Mann-Whitney rank sum test (giving median values) using SigmaStat 3.5 (Systat Software Inc., Chicago, IL, USA).

## Results

### Overall results and statistics

In order to identify early stages of disturbed bone turnover, we investigated the diagnostic value of routine assays for ALP activity and Ca and P_i_ levels in the plasma of mice derived from ENU-treated males for its comparability to their use in human patients (Table 1). This table also shows other mouse lines obtained for selected metabolic bone diseases and the observed alterations of plasma parameters in these models. Since we were interested only in mouse lines showing alterations of the bone ALP (bALP) isoform of the measured total ALP enzyme, variants with additional alterations of ALAT (alanine-amino-transferase) and ASAT (aspartate-amino-transferase) levels were excluded from this study.

Two hundred fifty-seven phenodeviants (2.7 %, 87 females and 170 males) of 9,540 F1 animals showed alterations in at least one of the three parameters of interest (ALP, Ca, and P_i_) in two repeated blood measurements. One hundred ninety of the 257 (74 %) phenodeviants were mated to wild-type C3H mice in confirmation crosses. In 71 of the mated 190 (37 %) (25 females and 46 males), the observed phenotype was genetically transmitted as a dominant trait (Table 2); however, six of these mutant lines were lost because no mutant male offspring was produced for sperm cryopreservation. For 110 of the mated 190 (58 %) phenodeviants, inheritance could not be confirmed because of sterility (*n* = 22/110, 20 %), the mice died due to unknown reasons (*n* = 15/110, 14 %), or the hypothesis of a dominant mutation was excluded (*n* = 73/110, 66 %). Confirmation crosses for the remaining 9 of the 190 phenodeviants are still underway. Sixty-seven of the 257 (26 %) phenodeviants were not mated due to space limitations; however, their sperm was frozen. Founder F1 mice with a similar phenotype and derived from the identical G0 male were expected to carry the identical mutation. Fifteen mutations have been identified resulting in new alleles of the *Phex*, *Casr*, and *Alpl* genes (Table 3).

**Table 2:** Genetic confirmation crosses and confirmed mutations for F1 variants with alterations of ALP activity and/or Ca and P_i_ plasma values

Phenotype^a^			Confirmation crosses		
ALP	Ca	P_i_	Total number	Confirmed (% of total F1 tested for this phenotype)	Ongoing
↑	Ø	Ø	83	28 (33.7)	1
↑	↑	Ø	4	2 (50)	0
↑	↑	↑	1	0	0
↑	↑	↓	1	1 (100)	0
↑	↓	Ø	1	1 (100)	0
↑	Ø	↓	14	4 (28.6)	1
↓	Ø	Ø	10	6 (60)	1
↓	Ø	↓	1	0	0
Ø	↑	Ø	20	9 (45)	0
Ø	↑	↑	1	0	0
Ø	↑	↓	8	4 (50)	2
Ø	↓	Ø	3	1 (33)	0
Ø	Ø	↑	2	1 (50)	0
Ø	Ø	↓	41	14 (34.1)	4
Total			190	71 (37.4)	9

*↑* high, *↓* low, *Ø* unchanged, *ALP* alkaline phosphatase, *Ca* total calcium, *P*_*i*_ total inorganic phosphate

**Table 3:** Confirmed mutant mouse lines with alterations of ALP activity and Ca and P_i_ plasma values

Line name	Variant phenotype	Additional phenotype, comment	Transmission (%)^a^
BAP001	High ALP		<20
BAP002	High ALP, high Ca	All variants with brittle teeth, jaw abnormality (~3 months old); changes in the tubular bone structure, reduced bone density	64
BAP003	High ALP, high Ca	Mapped on Chr 4 between SNP markers rs28307021 and rs3711383 (101.16–141.90 Mb, mouse genome Build 37.1, UCSC)	62
BAP004	High ALP and/or high Ca and/or low P_i_	All variants with auricle degeneration when >4 months old; reduced body size; mapped on Chr 4 between SNP markers rs28056583 and rs13469808 (86.81–117.55 Mb, mouse genome Build 37.1, UCSC)	75
BAP005	High ALP	Mutation of the *Asgr1* (asialoglycoprotein receptor 1) gene, c.815A > G; p.Tyr272Cys	92
BAP006	High ALP	Identical G0 as BAP007	lost
BAP007	High ALP	Identical G0 as BAP006; counted with BAP006 as one line	lost
BAP008	High ALP	Identical G0 as BPL001	71
BAP009	High ALP	All variants show circling behavior, reduced body size	32
BAP010	High ALP		lost
BAP011	High ALP		32
BAP012	High ALP, low P_i_	All variants small with shortened hind limbs, circling behavior. Nonsense mutation in exon 2 of the *Phex* (phosphate-regulating gene with homologies to endopeptidases on the X chromosome) gene, c.148A > T; p.Lys50X	100
BAP013	High ALP		27
BAP014	High ALP	Significantly more males born and affected; offspring of heterozygous intercrosses with reduced body size, ALP very high; mapped on Chr 9 between SNP markers rs3023207 and rs3673055 (37.50–96.23 Mb, mouse genome Build 37.1, UCSC)	60
BAP015	High ALP		lost
BAP016	High ALP		70
BAP017	High ALP	All variants with reduced body size	<20
BAP018	High ALP		96
BAP019	High ALP		29
BAP020	Low ALP	Synonymous sequence variation in exon 10 of the *Alpl* (alkaline phosphatase, liver/bone/kidney) gene, c.1098A > T, p.Thr365Thr	100
BAP021	High ALP	High ALAT and ASAT, phenotype probably liver dependent	44
BAP022	High ALP		100
BAP023	Low ALP	Missense mutation in exon 7 of the *Alpl* (alkaline phosphatase, liver/bone/kidney) gene, c.755T > G; p.Leu251Pro. Identical G0 animal as BAP021 and BCH009	100
BAP024	High ALP, low P_i_	All variants with reduced body size, circling behavior. Missense mutation in exon 22 of the *Phex* (phosphate-regulating gene with homologies to endopeptidases on the X chromosome) gene, c.2197T > C; p.Cys733Arg	100
BAP025	High ALP		100
BAP026	Low ALP	Splice site mutation in intron 9 of the *Alpl* gene (alkaline phosphatase, liver/bone/kidney) gene c.997+2T > G	85
BAP027	Low ALP	Missense mutation in exon 10 of the *Alpl* (alkaline phosphatase, liver/bone/kidney) gene, c.1194T > A, p.Ile395Asn	100
BAP028	High ALP	All mutants with reduced body size	100
BAP029	High ALP		30
BAP030	High ALP	All mutants with reduced body size	56
BAP031	High ALP		52
BAP032	Low ALP	Missense mutation in exon 11 of the *Alpl* (alkaline phosphatase, liver/bone/kidney) gene, c.1217A > G, p.Asp406Gly	90
BCH001	High Ca		<20
BCH002	High Ca, low P_i_	Missense mutation in exon 7 of the *Casr* (calcium-sensing receptor) gene, c.2579T > A; p.Ile859Asn. Some intercrosses derived offspring with reduced body size, gray fur	100
BCH003	High Ca	Missense mutation in exon 3 of the *Casr* (calcium-sensing receptor) gene, c.295G > T, p.Asp99Tyr. Identical G0 as BCH006	100
BCH004	High Ca	Nonsense mutation in exon 4 of the *Casr* (calcium-sensing receptor) gene, c. 366G > T, p.Glu456X	93
BCH005	High Ca		22
BCH006	High Ca	Identical G0 as BCH003; counted with BCH003 as a single line	100
BCH007	High Ca	Missense mutation in exon 4 of the *Casr* (calcium-sensing receptor) gene, c.626T > C; p.Val208Ala. Some intercrosses derived offspring show reduced body size.	74
BCH008	High Ca		78
BCH009	High Ca	Identical G0 animal as BAP021 and BAP023	<20
BCH010	High Ca		20
BCH011	High Ca	Nonsense mutation in exon 7 of the *Casr* (calcium-sensing receptor) gene, c.2017C > T, p.Gln673X	100
BCH012	High Ca		<20
BCH013	High Ca	Missense mutation in exon 3 of the *Casr* (calcium-sensing receptor) gene, c.296A > G, p.Asp99Gly	100
BCL001	Low Ca, high ALP		100
BCL002	Low Ca		50
BPH001	High P_i_		lost
BPL001	Low P_i_	Identical G0 animal as BAP008	<20
BPL002	Low P_i_	Mapped on Chr 16 between SNP markers rs4186801 and rs4199268 (51.47–69.80 Mb, mouse genome Build 37.1, UCSC)	38
BPL003	Low P_i_		<20
BPL004	Low P_i_	Mapped on Chr 3 between SNP markers rs13477178 and rs13477321 (69.55–109.00 Mb, mouse genome Build 37.1, UCSC)	63
BPL005	Low P_i_		46
BPL006	Low P_i_	All mutants with reduced body size; mapped on Chr 14 between SNP markers rs30406796 and rs30865397 (22.92–74.08 Mb, mouse genome Build 37.1, UCSC)	73
BPL007	Low P_i_	All mutants with reduced body size	53
BPL008	Low P_i_	All mutants with reduced body size; mapped on Chr 8 between SNP markers rs13479952 and rs13479998 (103.43–116.69 Mb, mouse genome Build 37.1, UCSC)	37
BPL009	Low P_i_		40
BPL010	Low P_i_		100
BPL011	Low P_i_		67
BPL012	Low P_i_		50
BPL013	Low P_i_		50
BPL014	Low P_i_		100
SAP003	High ALP		<20
SAP004	Low Ca, low P_i_		<20
SAP005	High ALP		lost
SAP006	High ALP		41
SAP007	Low ALP	Missense mutation in exon 12 of the *Alpl* (alkaline phosphatase, liver/bone/kidney) gene, c.1357A > G; p.Thr453Ala	100
SAP008	High ALP		62
SCA001	High Ca		49
SMA010	High ALP, high Ca	All variants with reduced body size (*Z* score < –2)	31
TRE002	High ALP	All mutants trembling, high ALP probably secondary effect	100

All mouse lines listed in alphabetical order of the internal lab names

^a^According to dominant inheritance 50 % mutant offspring corresponds to 100 % transmission of the phenotype

### New mouse lines carrying mutations of the *Phex* (phosphate-regulating gene with homologies to endopeptidases on the X-chromosome) gene

**Fig. 1:**
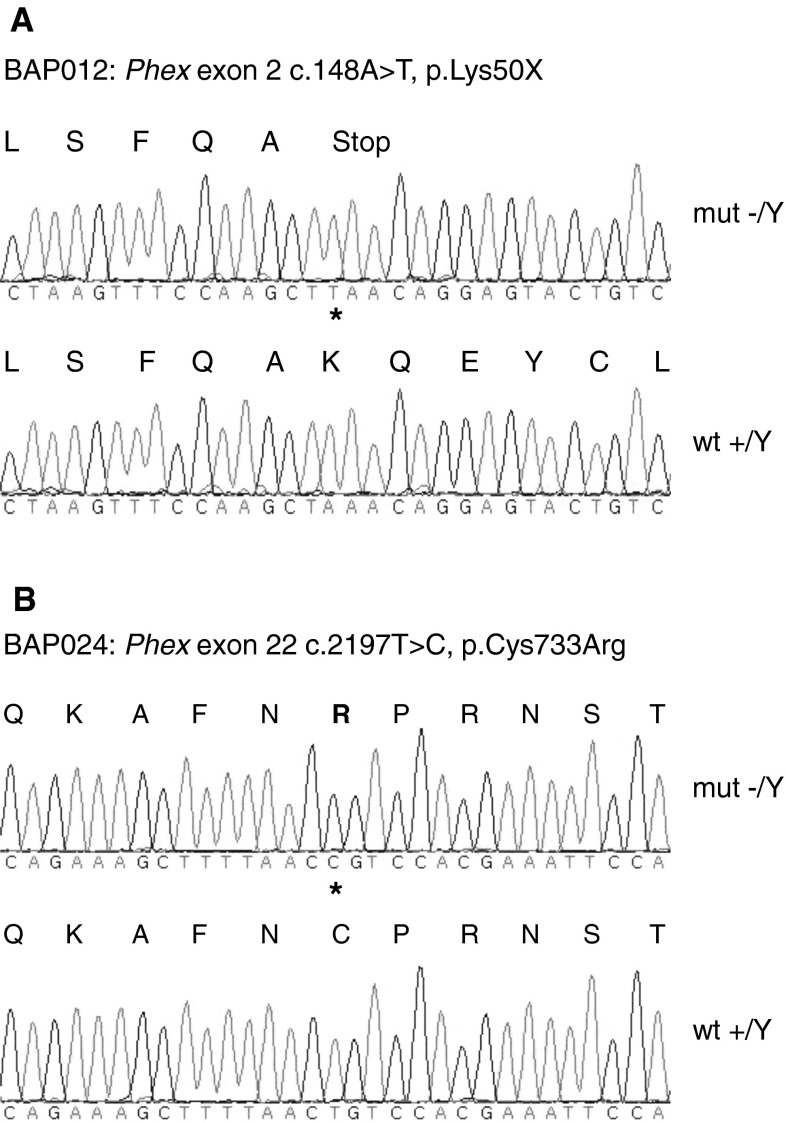
**a** DNA sequence analysis of the *Phex* gene exon 2 revealed a hemizygous nonsense mutation (c.148A > T, p.Lys50X) in the DNA of a male C3Heb/FeJ-Phex^BAP012^ mutant mouse leading to a premature translation stop codon after 49 amino acids. **b** DNA sequence analysis of the *Phex* gene exon 22 revealed a hemizygous missense mutation (c.2197T > C, p.Cys733Arg) in the DNA of a male C3Heb/FeJ-Phex^BAP024^ mutant mouse. Variants are marked by an *asterisk*

Affected animals of the BAP012 (Bone screen Alkaline Phosphatase No. 012) mutant line displayed a significant (*P* ≤ 0.001) decrease in plasma P_i_ levels. Female mutant mice (*n* = 42) exhibited a P_i_ value of 1.3 ± 0.2 mmol/l compared to female wild-type mice (2.0 ± 0.3 mmol/l, *n* = 11). Male mutant mice (*n* = 7) had a P_i_ value of 1.2 ± 0.1 mmol/l compared to 2.0 ± 0.3 mmol/l in male wild-type mice (*n* = 44). Mean ALP activity was significantly elevated (*P* ≤ 0.001) in female mutants (266.4 ± 35.3 U/l) compared to wild-type littermates (147.9 ± 17.9 U/l), and also in mutant male mice (370.9 ± 88.5 U/l) compared to their wild-type littermates (120 ± 8.5 U/l). In addition to these biochemical alterations, all mutants showed reduced body size, shortened hind limbs, and mild head-tossing behavior as described in other *Phex* mouse models (Lorenz-Depiereux et al. 2004; Moriyama et al. 2011). Genetic crosses revealed X-linked inheritance of the phenotype. Thus, mutant mice of both sexes were derived from mated mutant females, but from matings of male mutants only female mutants were born. Based on the phenotypic data, the causative mutation was hypothesized to be in the *Phex* gene. DNA sequence analysis of the *Phex* gene revealed a new hemizygous nonsense mutation in exon 2 (c.148A > T, p.Lys50X) (Fig. 1a). The mutation is located within the large extracellular domain of the protein close to the transmembrane domain. The *Phex* gene in mice is syntenic to the human *PHEX* gene, which is organized into 22 exons and encodes a type II transmembrane protein with homology to zinc metallopeptidases (HYP Consortium 1995). Inactivating mutations of the *PHEX* gene cause X-linked dominant hypophosphatemic rickets (XLHR), which has an incidence of 1:20,000 and is the most common familial form of hypophosphatemic rickets in humans (Burnett et al. 1964; Tenenhouse 1999).

Mice of the BAP024 mutant line express similar phenotypes, with gender influences on inheritance as the C3Heb/FeJ-Phex^BAP012^ mice. In BAP024 we found a new missense mutation in exon 22 of the *Phex* gene (c.2197T > C, p.Cys733Arg) (Fig. 1b), also located in the large extracellular catalytic domain of the protein. The cysteine at position 733 is highly conserved among other vertebrate species (Du et al. 1996). A cysteine-to-serine substitution at the corresponding position of the C3Heb/FeJ-Phex^BAP024^ mutation has been described recently in a patient with XLHR (Filisetti et al. 1999). No spontaneous *Phex* point mutations on the C3H strain have been isolated previously.

### New mouse lines carrying mutations of the *Casr* (calcium-sensing receptor) gene

**Fig. 2:**
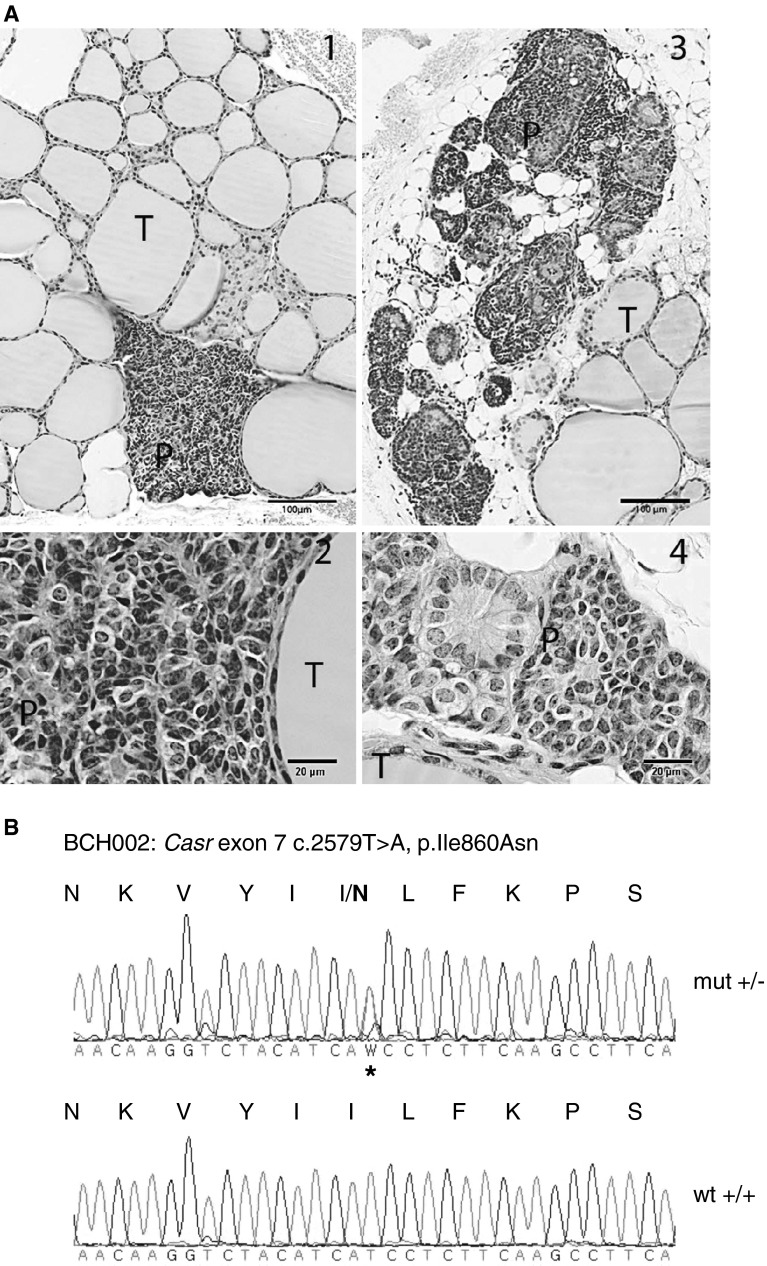
**a** Representative pictures of the histological changes found in the mutant mouse line BCH002: **1**, **2** (*left*) depict a H&E-stained section of normal thyroid gland (T) and parathyroid gland (P) in a control mouse. In **1** (low-magnification panel, 5×, scale bar = 100 μm), the normal gland appears as a small compact mass of dark cells. In **2** at higher magnification (20×, scale bar = 20 μm), two cell types interspersed with capillaries and sinusoids are identified: the chief cells, with a small basophilic cytoplasm, and the light cells, with abundant light cytoplasm. **3**, **4** (*right*) show a H&E-stained section of the normal thyroid gland (T) and the enlarged parathyroid gland (P) with loose structure observed in heterozygous C3HeB/FeJ-Casr^BCH002^ mutant mice (**3**). At higher magnification in **4**, an increase in the number of light cells is observed. **b** DNA sequence analysis of the *Casr* gene of C3HeB/FeJ-Casr^BCH002^ mice revealed a heterozygous mutation in exon 7 (c.2579T > A, p.Ile859Asn) that is marked by an *asterisk*

The BCH002 (Bone screen Calcium High No. 002) line showed a statistically significant increase of Ca levels in mutant animals compared to wild-type littermates (*P* ≤ 0.001). Female mutants’ Ca level was 2.9 ± 0.1 mmol/l (*n* = 23) compared to 2.43 ± 0.1 mmol/l for wild-type littermates (*n* = 19). The male mutant value was 2.87 ± 0.1 mmol/l (*n* = 20) compared to the wild-type littermates’ value of 2.41 ± 0.1 mmol/l (*n* = 20). Fifty-three percent of female and male mutant BCH002 mice had slightly reduced P_i_ levels. Histological analysis showed enlarged parathyroid glands in heterozygous mutant mice (Fig. 2a). A group of 11 female (6 mutants, 5 wild types) and 20 male mice (10 mutants, 10 wild types) was tested for PTH values, resulting in significantly raised median PTH values for mutant mice (*P* ≤ 0.001): female mutants, 214.9 pg/ml (25 % 203.3 pg/ml and 75 % 265.7 pg/ml), and wild types, 85.7 pg/ml (25 % 79.6 pg/ml and 75 % 113.1 pg/ml). Male mutants showed 235 pg/ml (25 % 191 pg/ml and 75 % 409.9 pg/ml) compared to wild types showing 102.7 pg/ml (25 % 78.8 pg/ml and 75 % 117.2 pg/ml). So far eight pups were derived from a first heterozygous intercross but no homozygous mutant was found. Mapping analysis of 40 mutant and 20 wild-type BCH002 animals derived from the dominant backcrosses to the B6 strain revealed linkage to chromosome 16 (Table 4), with the highest χ^2^ value at the marker rs4186801 (51.47 Mb, mouse genome Build 37.1, UCSC). In this region *Casr* was the most promising candidate gene for the observed phenotype. DNA sequence analysis of the *Casr* gene revealed a new heterozygous missense mutation (c.2579T > A, p. Ile859Asn) within the protein-coding region of exon 7 (Fig. 2b) of the gene that was not present in wild-type C3H and B6 mice. CASR belongs to the family of G-protein-coupled receptors (GPCRs) and is an integral membrane protein that senses changes in the extracellular calcium concentration to parathyroid cells.

**Table 4:** Statistical analysis of SNP mapping data of the C3HeB/FeJ-Casr^BCH002^ mutant line obtained by MyGenotype database

Chromosome (marker)	Highest χ^2^	Highest -log10(P)
1 (rs31593281)	5.57	1.74
2 (rs3691120)	1,80	0.75
3 (rs3685081)	1.72	0.72
4 (rs28307021)	0.78	0.42
5 (rs32067291)	3.60	1.24
6 (rs13478606)	3.76	1.28
7 (rs13479476)	1.09	0.53
8 (rs13479998)	8.02	2.34
9 (rs3023207)	5.00	1.60
10 (rs13480484)	4.45	1.46
11 (rs27000576)	9.38	2.66
12 (rs6194112)	7.36	2.18
13 (rs29566800)	4.45	1.46
14 (rs30482696)	2.17	0.85
15 (rs13482484)	9.38	2.66
16 (rs4186801)	28.17	6.96
17 (rs13483097)	4.79	1.54
18 (rs13483484)	0.82	0.44
19 (rs6339594)	2.69	1.00

In addition, six new alleles of the *Casr* gene were isolated in other mouse lines (BCH003, BCH004, BCH007, BCH011, BCH013, and BCH014) (Table 3) creating an allelic series for functional analysis of the gene. Median PTH levels were significantly increased (*P* *=* 0.010) in first testings of BCH013 female mutants (*n* = 6), showing 175.2 pg/ml (25 % 143.21 pg/ml and 75 % 198.33 pg/ml) compared to wild types (*n* = 4) showing 50.061 pg/ml (25 % 48.805 pg/ml and 75 % 56.962 pg/ml). Male mutants (*n* = 10) displayed 100.984 ± 30.765 pg/ml and wild types (*n* = 9) 55.485 ± 14.734 pg/ml. For all other mouse lines with mutations of the *Casr* gene, PTH data are underway. The missense and nonsense mutations of these mouse lines were located in exons 3, 4, 5, and 7 of the *Casr* gene (Table 3).

### New mouse lines carrying mutations of the *Alpl* (alkaline phosphatase, liver/bone/kidney) gene

**Fig. 3:**
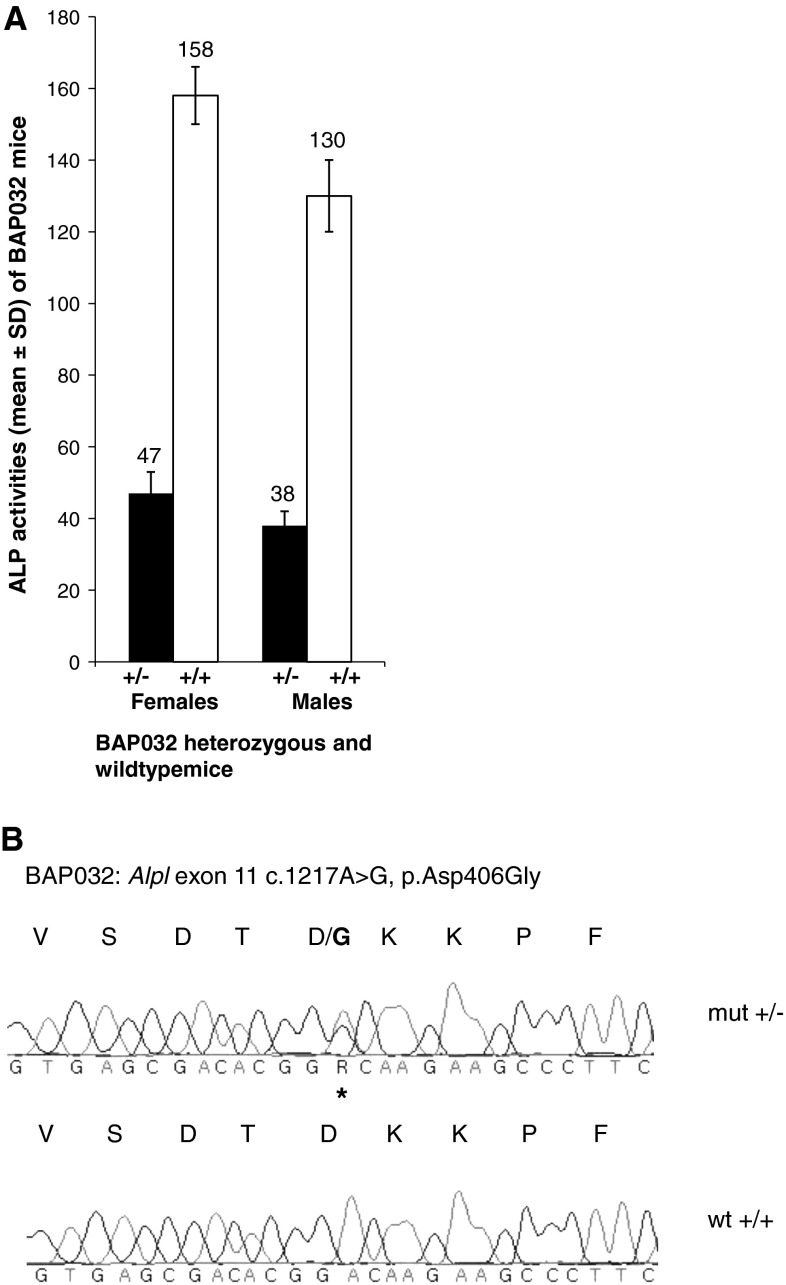
**a** C3HeB/FeJ-Alpl^BAP032^ ALP blood activities (mean ± SD U/l) in female mutant (*N* = 8), female wild-type (*N* = 9), male mutant (*N* = 12), and male wild-type (*N* = 10) mice. Mean ± SD ALP activities were: female mutants 47 ± 5.8 U/l (*P* < 0.001); female wild-types 157.8 ± 7.9 U/l; male mutants 38.4 ± 6.3 U/l (*P* < 0.001); male wild-types 129.5 ± 10.1 U/l (*t*-test). **b** DNA sequence analysis of the *Alpl* gene exon 11 revealed a new heterozygous missense mutation (c.1217A > G, p.Asp406Gly). Variant is marked by an *asterisk*

In mutant mice of the BAP032 line, statistically significant (*P* < 0.001) low mean ALP activity was found in female mutants (47 ± 5.8 U/l, *n* = 9) compared to wild-type littermates (157.8 ± 7.9 U/l, *n* = 8), and in male mutants (38.4 ± 6.3 U/l, *n* = 12) compared to wild-type littermates (129.5 ± 10.1 U/l, *n* = 10) (Fig. 3a). Significantly reduced ALP activity suggested a mutation in the *Alpl* gene encoding the tissue nonspecific ALP (TNSALP). We sequenced this gene in BAP032 mice and revealed a new heterozygous missense mutation in exon 11 located within the protein-coding region of the *Alpl* gene on chromosome 4 (c.1217A > G, p.Asp406Gly) (Fig. 3b). This mutation was not found in wild-type C3H littermates or in wild-type B6 mice. We isolated five additional mouse lines carrying new alleles of the *Alpl* gene (Table 3). Four sequence variations were located in exons 7, 10, or 12 (BAP020, BAP023, BAP027, SAP007) and one affects the splice site in intron 9 (BAP026).

### Other mouse lines and mutations

Female and male mutant mice of the BAP005 line showed statistically significant (*P* ≤ 0.001) increased mean ALP activity. The value in female mutants (*n* = 25) was 233 ± 21 U/l compared to 136 ± 14 U/l in wild-type littermates (*n* = 32), and in male mutants (*n* = 39) ALP activity was 188 ± 19.81 U/l compared to 104.5 ± 10.43 U/l in their wild-type littermates (*n* = 36). The mouse line breeds homozygous offspring with very high ALP activities. Homozygous females derived from heterozygous intercrosses showed mean ALP activity of 587 ± 39 U/l (*n* = 12), and ALP activity in homozygous males was 482 ± 51 U/l (*n* = 21). SNP mapping revealed a region between the markers rs26982471 and rs27000576 (53.99–114.33 Mb, mouse genome Build 37.1, UCSC) on chromosome 11. Sorting of chromosome 11 and whole chromosome 11 sequencing on a GAIIx next-generation sequencing machine revealed a new missense mutation in the *Asgr1* (asialoglykoprotein receptor 1) gene within the translated region (c.815A > G, p.Tyr272Cys). The mutation was sequenced in 16 BAP005 mutant mice, but neither in 4 wild-type littermates, nor in additional 4 wild-type mice from different inbred strains (BALB/c, DBA/2, FVB, SJL).

For eight additional mouse lines (BAP002, BAP003, BAP004, BAP014, BPL004, BPL006, BPL008, and TRE002) showing high ALP activity, low P_i_, and high or low Ca values as a phenotype, genetic mapping has been finished (Table 3) and sequencing of candidate genes is in progress. For selected mouse lines we will include exome sequencing to find the causative mutation.

## Discussion

In this study we described a large-scale ENU mutagenesis screen (Soewarto et al. 2009), with the main focus on malfunctioning bone turnover. In other projects murine models for disturbed bone metabolism were obtained by gene targeting (Daroszewska et al. 2011; Ducy et al. 1996; Forlino et al. 1999; Kato et al. 2002), transgene insertions (Imanishi et al. 2001; Rauch et al. 2010) or spontaneous mutations (Eicher et al. 1976; Marks and Lane 1976). Here, we isolated 71 new mouse models by screening for alterations of total ALP activity and total Ca and P_i_ values in plasma of 9,540 F1 mice. Our results demonstrate that malfunctions of bone metabolism in mice may be efficiently detected by the analysis of human standard clinical chemical parameters.

In this study the highest fraction of new mouse lines revealed alterations of total ALP activity (Table 3). Since these mouse lines discriminated in phenotype expression and occurrence of additional phenotypes, the phenotypes presumably depend on different molecular mechanisms. Total ALP was chosen as a parameter of interest since elevated ALP activity is the most frequently measured parameter for human Paget’s disease (Langston and Ralston 2004), X-linked hypophosphatemic rickets (XLHR) (Jonsson et al. 2003; Mäkitie et al. 2003), autosomal dominant hypophosphatemic rickets (ADHR) (Econs and McEnery 1997; Imel et al. 2007; Kruse et al. 2001), and type I osteoporosis (Avbersek-Luznik et al. 2007; Pedrazzoni et al. 1996).

Screening for alterations of total Ca and P_i_ values without changes of ALP activity resulted in 34 mutant lines with confirmation of the observed phenotype (Table 2). While the Ca parameter was easy to measure, P_i_ values were artificially elevated after plasma storage for longer than 1 day, freezing of the samples, or hemolysis. Metabolic bone diseases may be reflected in changes of more than one parameter, and very often two or three of the parameters of interest showed alterations in the same individual mouse line, as is commonly observed in human patients (Table 1).

In our screen we obtained new mouse models for hypophosphatemia, hyperparathyroidism, and hypophosphatasia. Despite the large number of existing mouse models for XLHR, there are still open questions on the mechanism of PHEX in renal phosphate wasting, abnormal vitamin D metabolism, and matrix mineralization (Addison et al. 2010; Brownstein et al. 2010). The C3HeB/FeJ-Phex^BAP012^ and C3HeB/FeJ-Phex^BAP024^ mutant lines represent two new mutant mouse lines with novel point mutations modeling XLHR in addition to previously published models (Carpinelli et al. 2002; Lorenz-Depiereux et al. 2004; Xiong et al. 2008).

New point mutations of the *Casr* gene were found in seven mouse lines. The large extracellular domain of the receptor contains clusters of amino acid residues, which may be involved in calcium binding (Brown et al. 1993). Exon 7 encodes the seven transmembrane domains and four intracellular loops of CASR (Chang et al. 2008). Human *CASR* mutations are known to be causative for primary hyperparathyroidism (HP) (Bilezikian et al. 2005) and familial benign hypocalciuric hypercalcemia (FHH) (Pollak et al. 1994). Approximately two thirds of FHH patients showed loss-of-function mutations involving the 3,234-bp coding region of the *CASR* gene (D’Souza-Li et al. 2002). Individuals with HP and FHH discriminate in creatinine clearance and serum magnesium values, both being higher in FHH (Marx et al. 1981). It has been demonstrated that individuals with FHH are heterozygous, and children within these families with severe neonatal primary hyperparathyroidism (NSHPT) are homozygous for *CASR* mutations (Janicic et al. 1995; Pollak et al. 1993). Mice with tissue-specific deletion of *Casr* in the parathyroid gland and bone exhibited profound bone defects (Chang et al. 2008). C3H;102Casr^Nuf^/H mice carry an activating ENU-derived *Casr* point mutation that exhibits hypocalcemia, hyperphosphatemia, cataracts, and ectopic calcifications (Hough et al. 2004). We obtained the first presumed loss-of-function point mutation isolated in C3HeB/FeJ-Casr^BCH002^ mice that is supposed to model human FHH. Since the mouse line has been bred more than 15 generations and because we found six other independent *Casr* mutations for this phenotype, it is more than likely that the consistent phenotype is due to the isolated point mutation of the *Casr* gene. Heterozygous C3HeB/FeJ-Casr^BCH002^ mice exhibited high Ca and PTH values similar to targeted Black Swiss/129SvJ *Casr* ^+/−^ and *Casr* ^−/−^ mice (Ho et al. 1995), but, in addition, they showed enlarged parathyroid glands described only for *Casr* ^−/−^ mice. Further heterozygous intercrosses are required to find out if homozygous C3HeB/FeJ-Casr^BCH002^ mice are viable, which is not the case in *Casr* ^−/−^ mice. This would raise the opportunity to obtain a mouse model for NSHPT. More than 270 mutations have been described so far in the human *CASR* mutation database (www.casrdb.mcgill.ca; Nakajima et al. 2009), and interestingly most of the human mutations were found in exons 4 and 7. The mouse lines carrying *Casr* mutations obtained in our screen showed slight differences in the expression of the phenotype. Additional studies on phenotypical and histological traits will help to discriminate between the different effects of each point mutation on the severity of hyperparathyroidism and concomitantly to improve our understanding of *CASR* mutations in human patients.

Heterozygous C3HeB/FeJ-Alpl^BAP032^ mice showed a statistically significant reduction of ALP activity in plasma without additional phenotypes, as observed in heterozygous *Akp2* ^*Hpp*/+^mice derived in an ENU mutagenesis screen on C3H/HeH background (Hough et al. 2007). In *Akp2* ^*Hpp*/+^ mice, an *Alpl* loss-of-function mutation led to the rare disease hypophosphatasia (HPP) which displays reduction of plasma ALP activities to about 50 % in *Akp2* ^*Hpp*/+^ and a stronger reduction in *Akp2* ^*Hpp*/*Hpp*^ mice. *Akp2* ^*Hpp*/+^ mice were radiographically and histologically indistinguishable from wild-type mice at different time points, as were 16-week-old C3HeB/FeJ-Alpl^BAP032^ mice in DEXA and X-ray analysis. Interestingly, we observed a stronger ALP reduction in heterozygous C3HeB/FeJ-Alpl^BAP032^ mice than in *Akp2* ^*Hpp*/+^mice, with ALP activities in female and male mutant mice reduced to 29 % of that found in wild-type littermates. Severe HPP forms are characterized by hypomineralization, rickets, seizures, and nephrocalcinosis due to hypercalciuria (Beck et al. 2009). *Alpl* ^−/−^ mice showed a reduction in body size, no detectable ALP levels, and lethality prior to weaning, whereas *Alpl* ^+/−^ mice appeared healthy (Narisawa et al. 1997). The identical point mutation of C3HeB/FeJ-Alpl^BAP032^ mice has also been described for a patient with HPP (Taillandier et al. 2000). Heterozygous C3HeB/FeJ-Alpl^BAP032^ mice presumably model mild adult HPP. The mouse line was bred for more than ten generations, showing full penetrance of the phenotype in all litters. A multitude of diverse point mutations, deletions, and insertions of the human *TNSALP* gene causing HPP are listed in the hypophosphatasia database (www.sesep.uvsq.fr/03_hypo_mutations.php). The diversity of published human point mutations emphasizes the importance of mouse models for further investigations on physiological functions and cellular mechanisms of *Alpl* regions involved in collagen and Ca binding. Interestingly, we isolated in addition one silent mutation in the BAP020 mouse line (Table 4) showing the expected phenotype. No additional *Alpl* mutations were found in this mouse line. *Alpl* mRNA and translation of ALP were not analyzed so far.

Since only total ALP can be tested in mice so far, we probably will isolate mouse lines showing alterations other than the bone ALP isoform. High alterations of plasma ALP activities without any additional phenotypes, as observed in homozygous animals of the C3HeB/FeJ-Asgr1^BAP005^ line, have not been published in mice before. It has been described in patients with chronic liver disease that the adult intestinal ALP isoenzyme was increased due to the reduced efficiency or numbers of asialoglycoprotein receptors (Moss 1994). Thus, the mutation of the gene in BAP005 mice seems to cause alterations of the intestinal ALP isoform as a secondary effect. *ASRG1* mutations may be responsible for high ALP activities of so far unknown reasons in humans without any skeletal disorders (Panteghini 1991) or may cause benign familial hyperphosphatasemia (Siraganian et al. 1989).

We have to consider bone as an active metabolic organ with a possible influence on metabolism in diseases of disturbed bone turnover (Ferron et al. 2010; Fulzele et al. 2010). For this reason, systematic analysis of all organ systems, as in the German Mouse Clinic (Gailus-Durner et al. 2005), might provide new insights into the actions in these pathways. Our mouse models will be archived by the European Mouse Mutant Archive (EMMA) and are available (www.emmanet.org) for the scientific community.
